# Medical Training in Uganda: A Critical but Neglected Part of the Healthcare System

**DOI:** 10.7759/cureus.40044

**Published:** 2023-06-06

**Authors:** Esau Ogei, Catherine Lewis

**Affiliations:** 1 Internal Medicine, St. Joseph's Hospital Kitovu, Masaka, UGA; 2 General Surgery, East Tennessee State University, Johnson City, USA; 3 General Surgery, St. Joseph's Hospital Kitovu, Masaka, UGA

**Keywords:** africa, uganda, strike, house officer, medical officer, medical internship, global healthcare systems

## Abstract

Quality healthcare is dependent upon the structure of healthcare and/or healthcare facilities in a country. In Uganda, the healthcare system has had drastic changes over the last 50 years. Medical students, interns, and medical officers play an invaluable role in the function of hospitals and the overall quality of the healthcare system of Uganda, particularly in government facilities. Demands for better working conditions and payment of arrears have forced the graduate medical students and upcoming medical interns to strike, causing disruption in the fulfillment of basic health services. In order to prioritize the care of patients in the country, there should be fair treatment of the medical workers to boost and maintain morale and ultimately lead to continued quality patient care.

## Editorial

Uganda's healthcare system infrastructure

In the 1960s, Uganda had one of the best healthcare systems in East Africa. Hospitals were well-equipped, well-staffed, and had a set of connected healthcare units. Political turmoil from 1970 to 1985 fragmented this healthcare system [1,2]. The Ministry of Health (MOH) of Uganda is responsible for the majority of the healthcare services. The private sector and non-governmental organizations (NGOs) also play an important role [2]. The Uganda healthcare system has a hierarchy of health centers and hospitals that consist of seven levels [1]. Level V refers to general hospitals that are in each district. They have specialized clinics and consultants, and many are in the private sector. Level VI refers to regional referral hospitals that have specialized services and some residency programs. Currently, there is one level VII national referral hospital. This facility has advanced diagnostic services, advanced research capabilities, and “super-specialists.” Uganda reintroduced free healthcare in the country in 2001. This free healthcare is at the regional and national referral hospitals in which the majority of interns and residents train [2]. Interns are also offered training at level V facilities.

Medical education in Uganda

Supervised medical work for one year is a mandatory stage for graduate medical doctors, pharmacists, dentists, and nurses before licensure. In Uganda, intern doctors, also known as junior house officers (JHOs) work with a provisional license under the supervision of senior medical doctors in the fields of surgery, internal medicine, pediatrics, and obstetrics and gynecology for one year. Thereafter, the new doctors are fully licensed by the Uganda Medical and Dental Practitioners Council (UMDPC). The current graduates who are waiting for internships (pre-interns) finished their education about a year ago and are awaiting deployment by the MOH of Uganda.

Senior house officers (SHOs) are qualified medical doctors who are registered and licensed by the UMDPC. However, they are undergoing postgraduate training to become specialists in various fields of medicine [3]. The JHOs and SHOs make up over 75% of the human resources for doctors in regional referral hospitals, national referral hospitals, and many private not-for-profit hospitals, and they are almost always the first responders [4]. They deliver more than 90% of emergency obstetrics care in regional and national referral hospitals where 78% of all maternal deaths occur [5].

Nationwide strike by medical interns

Because of their tremendous contribution to the workforce, it was resolved that JHOs and SHOs would be paid a monthly allowance of 2,500,000 Ugandan Shillings with effect from July 1, 2021 [5,6]. However, these allowances are often not paid on time, and currently, there is a proposition to not pay these doctors at all. The current first-year SHOs have not yet received any government allowances, while the second and third-year SHOs have not been paid for about four to six months now [3-5,7].

On April 5, 2023, an announcement was made by the Honorable Minister of Health that there would be a delayed deployment of the JHOs for the year 2023-2024. This was after a wait of over nine months for those who had completed final exams in May 2022. Later, an announcement was made that subsequently there would be no payment for their work and that those who could not pay their way through internship would therefore not receive their medical licenses. The internship year would be considered a mandatory extra year for a medical degree, which is contrary to the current standard [4]. The presentation of the MOH budget did not include JHO and SHO payments despite having had their monthly allowances raised less than two years ago.

Following the delayed deployment of JHOs, the pre-interns took to the streets to protest. Many of them were inhumanely treated and arrested by the Uganda Police Force (UPF) [4]. Meanwhile, the SHOs reactivated their industrial action and laid down their tools, and ceased working on May 1, 2023, over the payment of arrears [3,5].

Why medical interns strike

Healthcare worker strikes have become a growing concern to the international health community. The World Health Organization (WHO) mentions “a sufficient capacity of well-trained, motivated health workers” as a component of achieving universal health coverage. The inability of governments to deliver necessary healthcare quality and contribute to a better life for their citizens has led to healthcare worker strikes in low and middle-income countries such as Zimbabwe, Haiti, Kenya, South Africa, Nigeria, Sudan, and Uganda [8]. Low pay or no pay has led to the majority of the strikes in Uganda [8-10]. The payment of monthly allowances of JHOs and SHOs in Uganda is frequently delayed, forcing many to live in harsh conditions. They may live in poor housing or be evicted from houses they rent. While hospitals in rural areas may be able to provide accommodations, over 100 interns living in the hostel of the national referral hospital were evicted for renovations in 2014. Interns in the public government hospitals always must find accommodation, food, and transportation, which is difficult when there is a delay or no payment [10].

Interns are oftentimes forced to work over 12 hours a day, seven days a week, without days off [10]. During the coronavirus disease 2019 (COVID-19) pandemic and the recent Ebola outbreak, many doctors worked long hours, were forced to quarantine, and sometimes worked without protective gear [11].

Medical interns are supposed to be fully supervised by a senior consultant. However, oftentimes interns work more closely with medical officers or SHOs. There is widespread absenteeism of senior consultants and a lack of oversight that also causes the JHOs and SHOs to strike. Medical officers have completed their internship but have not yet secured a spot as an SHO. They, therefore, do not have as much experience as the senior consultants [10].

Consequences of the medical healthcare worker strike in Uganda

The impact of healthcare worker strikes depends on the duration and location in which the strike occurs. In a low-income country such as Uganda, there is a greater impact on poor and vulnerable patients [8]. The current nationwide strike in Uganda has the potential to lead to doctors who are inadequately trained [11]. Further delay in training will ultimately lead to a shortage of well-trained doctors in Uganda as there will not be any new senior consultants who will enter the workforce. Further delay may also cause many doctors to pursue training in other countries, leading to a shortage of physicians in Uganda. Without the main source of healthcare providers, the country may experience increased mortality with the return of any outbreak or pandemic.

Conclusion

These endless strikes and issues of delayed deployment have always deterred service delivery in the regional and national referral hospitals, and even in private hospitals where interns train. This sad situation, which has negatively affected the entire healthcare workforce of Uganda, has inevitably contributed to a less-than-ideal healthcare service delivery system. Medical workers deserve to be paid for their hard work, and appreciated for their performance, particularly in low-resource settings. A boost in the morale of Uganda’s medical workers will ultimately lead to an overall improvement in the healthcare system.
